# Synthesis, Characterization, and Photocatalytic Activity of Ba-Doped BiFeO_3_ Thin Films

**DOI:** 10.3390/ma15030961

**Published:** 2022-01-26

**Authors:** Khiat Abdelmadjid, Felicia Gheorghiu, Boughelout Abderrahmane

**Affiliations:** 1 Research Center in Industrial Technologies (CRTI), P.O. Box 64 Cheraga, Algiers 16014, Algeria; khiatmadjid2018@gmail.com (K.A.); abderrahmanebough@gmail.com (B.A.); 2 RAMTECH, Department of Exact and Natural Sciences, Institute of Interdisciplinary Research, Alexandru Ioan Cuza University of Iasi, Blvd. Carol I, nr. 11, 700506 Iasi, Romania

**Keywords:** perovskites, films, optical properties, doped BaTiO_3_, photocatalytic activity

## Abstract

In the present paper, Bi_1−x_Ba_x_FeO_3_ (BBFO) thin films (where x = 0, 0.02 and 0.05) were prepared by a combined sol-gel and spin-coating method. The influence of Ba substitutions on the structural, microstructural, optical properties, and photocatalytic activity of BiFeO_3_ thin films has been studied. X-ray diffraction pattern correlated with FTIR analysis results confirms that all the films have a perovskite structure of rhombohedral symmetry with an R3m space group. Atomic force microscopy (AFM) and scanning electron microscopy (SEM) were used to investigate the surface morphology and reveals microstructural modifications with the increase in Ba concentration. The optical properties show that the band gap is narrowed after doping with Ba ions and decreases gradually with the increase of doping content. The photocatalytic investigations of deposited films revealed that Ba doping of BFO material leads to the enhancement of photocatalytic response. The present data demonstrates that Bi_1−x_Ba_x_FeO_3_ (BBFO) thin films can be used in photocatalytic applications.

## 1. Introduction

Today, evolution of modern society leads to a continuous increasing of environmental pollution. Therefore, degradation of organic pollutants shows a great interest for photocatalytic applications [1]. To date, several semiconductors can act as photocatalysts: TiO_2_, ZnO, Fe_2_O_3_, CdS, ZnS) [2,3,4,5,6]. TiO_2_ is the most studied photocatalysts [2,7,8], but the wide band gap (3.2 eV) [8] provides low efficiency of absorption in the visible-light region. This problem leads to the searching for new oxide-based photocatalysts with strong absorbance for visible- light applications. Semiconductor-based photocatalysts are promising materials for degradation of a large number of organic contaminants.

In the last years, the multiferroic materials have become an attractive research area due its interest in photocatalytic field [9,10]. The advantage of multiferroics in comparison with usual photocatalysts is their low band gap energy. Among multiferroic materials, perovskite BiFeO_3_ (BFO) is one of the few and best known room—temperature multiferroics, that has a distorted perovskite structure that belongs to rhombohedral *R3c* symmetry (with *a* = 3.958 and α = 89.30°), with a high ferroelectric Curie temperature (*T_C_* = 830 °C) and antiferromagnetic order (*T_N_* = 370 °C) [11,12,13]. The preparations of BiFeO_3_ powder material were reported by different synthesis methods, as for example: solid-state reaction method [14], hydrothermal synthesis route [15], sol-gel process [16] combustion method [17], Pechini method [18], and microwave synthesis [19]. However, the preparation of BiFeO_3_ is very difficult due to: (i) The kinetics of phase formation in the Bi_2_O_3_-Fe_2_O_3_ system that leads to the formation of impurities along with the perovskite major phase [20] and thermal stability; (ii) valence fluctuation of iron (Fe^3+^/Fe^2+^ transitions) which leads to the variation of oxygen stoichiometry and appearance of the oxygen vacancies as defects; (iii) Bi volatilization because bismuth oxide has a low melting point. Therefore, in spite of different preparation methods, the powdered photocatalysts presents some disadvantages in comparison with thin films: difficulty in particle separation, a high cost, agglomeration effect at high concentration [21]. Consequently, BiFeO_3_ thin films have drawn considerable interest due do its potential to be used as photocatalytic films.

In order to overcome the problems and limitations regarding the obtaining of BiFeO_3_ powder system, a strategy was adopted of using various dopants substitution on Bi sites as Ca, Sr, Ba, Nd, Sm, La [22,23,24,25]. In the present case, Ba^2+^ ion as dopant in BFO was chosen, as its ionic radius (1.35 Å) is higher than that of Bi^3+^ ion (1.03 Å) and can compensate the Fe-O-Fe exchanges. By Ba ion substitution on Bi sites, it is also expected that the surface area will increase and improve the photocatalytic activities by decreasing the band gap energy. In the literature Ba-doped BiFeO_3_ material as nanoparticles [26], nanofibers [27], ceramics [28], and even as thin films was reported [29,30]. However, there are no reports focused on the Ba-doped BiFeO_3_ thin films as photocatalysts.

In the present paper, Bi_1−x_Ba_x_FeO_3_ (BBFO) thin films (where x = 0, 0.02 and 0.05) were prepared by a combined sol-gel and spin-coating method. The influence of Ba substitution on the structural (using XRD analysis), morphology (by SEM and AFM investigations), and narrowing of the band gap energy was investigated and the obtained results are discussed in detail. The main goal of the present paper is the photocatalytic activity investigations, data that were not reported until this moment for Bi_1−x_Ba_x_FeO_3_ as thin films. The photocatalytic activity of BBFO thin films was investigated by the degradation of Rhodamine B (RhB) under solar light irradiation. Rhodamine B was chosen as the organic pollutant for this study because it is reported in the literature with high degradation efficiency [21]. Ba influences on the photocatalytic properties will be also discussed.

## 2. Preparation and Experimental Details

**Figure 1:**
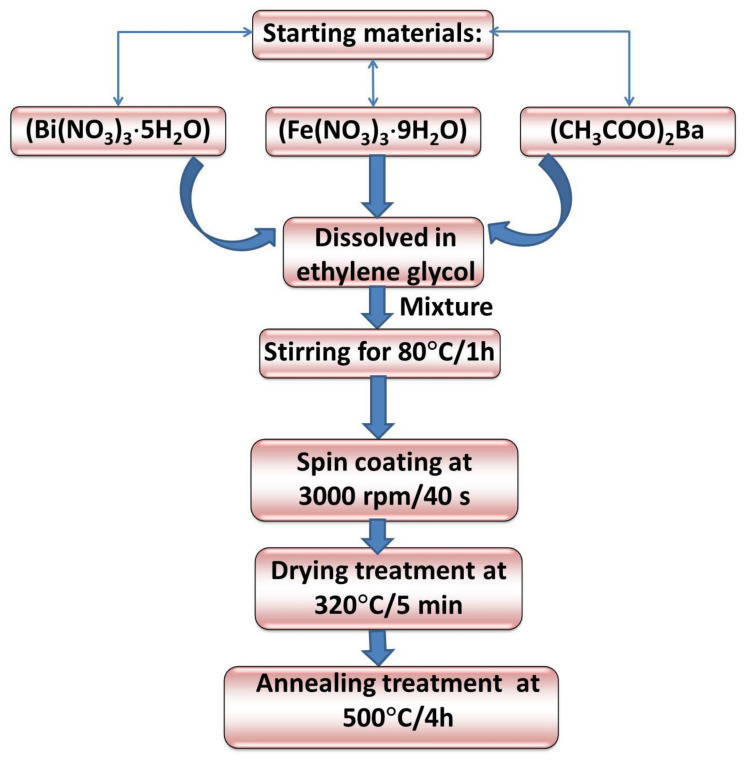
Preparation flowchart of Bi_1−x_Ba_x_FeO_3_ (BBFO) thin films.

BBFO thin films were synthesized on glass substrates by using sol-gel-assisted spin-coating technique, according to the preparation scheme represented in the Figure 1. Bismuth nitrate pentahydrate (Bi(NO_3_)_3_·5H_2_O, Sigma-Aldrich, Saint Louis, MO, USA), iron nitrate nonahydrate (Fe(NO_3_)_3_·9H_2_O, Sigma-Aldrich, Saint Louis, MO, USA), and barium acetate ((CH_3_COO)_2_Ba, 99%, Sigma-Aldrich, Darmstadt, Germany) were used as the starting precursors and were dissolved in ethylene glycol as solvent in order to obtain a final precursor solution of 0.2 M concentration.

The chosen Ba dopant concentration in BBFO thin films are as follows: x = 0, x = 0.02 and x = 0.05. In order to prevent and compensate for a possible volatilization of Bi during post deposition annealing processes, 10% excess bismuth nitrate was added. The resulted solutions were mixed together and then stirred for 1 h at 80 °C in order to get a well-mixed BBFO gel solution.

Then a few drops of acetic acid were added to the BBFO solution as a stabilizer. Afterwards, this mixed solution was spin-coated onto ultra-cleaned glass substrate, while spinning at a high speed of 3000 rpm for 40 s to obtain uniform films. After spinning, a drying treatment of 320 °C for 5 min is applied for removing the remaining solvent. The spin-coating and drying treatment processes are repeated twice. At the end, the films were transferred to the furnace and annealed at 500 °C for 4 h in ambient atmosphere to consolidate the films by a complete crystallization.

The phase formation of the BBFO thin films after the thermal treatment step were checked at room temperature with PANalytical Empyrean X-ray (Almelo, The Netherlands) diffractometer using CuKα radiation (λ = 1.5406 Å) and operating in the 2θ range of 20–60°, with a step size of 2θ = 0.04°. The phase identification was performed using X’pert High Score software (version 4.9) supported with ICDD database. The phase formation was checked also by using Fourier transform infrared (FTIR) spectra, recorded in the range 600–3000 cm^−1^ on a Bruker Vertex 70 FTIR Spectrometer with the aim to complete the phase identification by ascribing the peaks to the various modes.

The film’s surface images and morphologies were investigated by using a scanning electron microscope (SEM, type JEOL JCM-5000 Neoscope, Akishima, Tokyo, Japan) and by atomic force microscopy (AFM, type JEOL SPM5200, Tokyo, Japan). The UV-visible absorption spectra were recorded by using UV-vis spectrophotometer (Lambda 35, from Perkin Elmer, Waltham, MA, USA). The investigation of the prepared films as photocatalysts for degrading of organic RhB was carried out under natural sunlight with an irradiation power of ~800 mW/cm^−2^. Day light from a September sunny day with about 25 °C was used to perform the photocatalytic experiments. In order to prevent thermal effect or vaporization, the films were placed into a Pyrex reactor container. The reactor was placed for cooling into a thermostatic water bath during the measurements in order to maintain a constant temperature of 25 °C. In each experiment, a solution of RhB (100 mL volume, 10 mg/L concentration) was taken for batch studies.

## 3. Results and Discussion

### 3.1. Phase, Structural, and Microstructural Characterization

**Figure 2:**
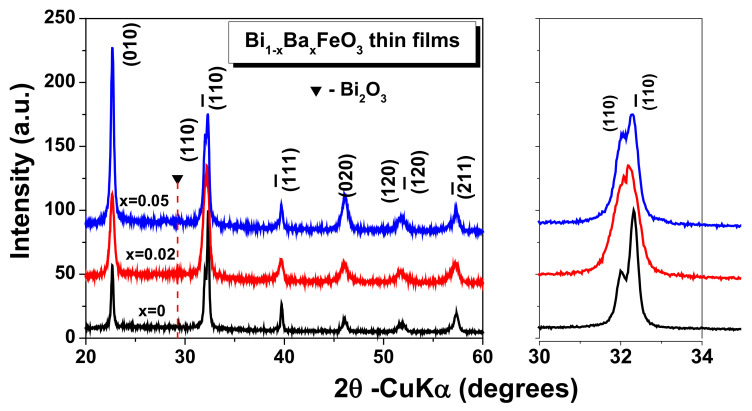
The XRD pattern for Bi_1−x_Ba_x_FeO_3_ (BBFO) thin films.

The XRD pattern for Bi_1−x_Ba_x_FeO_3_ thin films (where x = 0, 0.02 and 0.05) measured at room temperature is shown in Figure 2. The XRD peaks for x = 0 and 0.05 corresponding to the planes (010), (110), ( $\overset{-}{1}10$), ($\overset{-}{1}11$), (020), ($\overset{-}{1}20$), ($\overset{-}{2}11$), indicates the formation of single-phase perovskite with typical rhombohedral type structure [11,21,26,29,30] with the *R3m* space group identified with ICDS file no. 01-072-2112.

The XRD patterns confirm the incorporation of Ba^2+^ ions in the BiFeO_3_ lattice, with small traces of secondary phases of Bi_2_O_3_. Anyway, it can be observed from enlarger view of the XRD pattern within 2θ between 30° and 34° that doping with Ba^2+^ ions induces changes in structural stability of the pure BFO. A small increase in the dopant concentration leads to a slightly shift in the principal peak position to the lower angles than of the pure BFO, which can be associated to the strain relaxation in thin films with dopant additions. Present results are in good agreement with reported data from Ref. [26]. This small shifting can be explained by taking into account the higher value of the Ba^2+^ ionic radius in comparison with that one of Bi^3+^ which distorts the original structure of pure BFO due to the internal stress induced by Ba ions in the perovskite structure of BFO. The XRD intensities peaks and full width at half maximum intensity (FWHM) positions modification are the effect of the Ba addition and can be explained by considering the contributions from both the crystallite size and the strain that may be present in the films.

In order to understand the effect of Ba doping on the phase and structural, the crystallite size and tolerance factor were calculated and analyzed and the results are summarized in the Table 1. The crystallite size of the investigated Bi_1−x_Ba_x_FeO_3_ thin films has been calculated from XRD data using the Scherrer’s formula [31] as given by the following Equation (1):

(1) $$D=\frac{0.9\lambda }{\beta \cos\theta }$$

where *D* is the average crystallite size, 0.9 (constant) is a shape factor, *λ* = 1.5406 Å is the wavelength of used CuKα radiation, *β* is the peak broadening at half of the maximum intensity (FWHM, in radians) after subtracting the instrumental line broadening, and *θ* is the diffraction Bragg angle. In comparison with pure BFO, Ba^2+^ substitutions lead to a decrease of crystallite size in BBFO thin films.

To describe the stability and structural deformation of the perovskite structure, the Goldschmidt tolerance factor was calculated after the formula given by the Equation (2):

(2) $$t=\frac{\left(\left(1-x\right)r_{\mathrm{Bi}}{+\mathrm{xr}}_{\mathrm{Ba}}\right){+r}_{O}}{\sqrt{2}\left(r_{\mathrm{Fe}}{+r}_{O}\right)}$$

where *r_Bi_*, *r_Ba_*, *r_Fe_*, and *r_O_* are the effective ionic radii of Bi^3+^ (1.03 Å), Ba^2+^(1.35 Å), Fe^3+^ (0.65 Å), and O^2−^ (1.42 Å) ions respectively. The tolerance factor is calculated using the ionic sizes from Shannon [32]. From data reported in the Table 1, it can be observed that the tolerance factor increases slightly but gradually with Ba^2+^ ions concentration from 0.89 for the pure BFO to 0.893 for the BBFO thin films. It is known that for the ideal cubic perovskite structures, tolerance factor is about 0.9–1 value and for distorted perovskite structure (e.g., orthorhombic/rhombohedral) the values are between 0.71 and 0.9 [33]. According to the Equation (2), the fact that Ba^2+^ ionic radius is bigger than Bi^3+^ leads to an increase in tolerance factor which is in good agreement with the calculated results. Most probably, the Ba-doped BiFeO_3_ system is tending to transform from rhombohedral to a pseudo-cubic structure since the value of the tolerance factor for the highest Ba^2+^ concentration (x = 0.05) is tilting to 0.9 value. This also results in BFO covalent bonds changes, including bond angles like Fe-O-Fe, Bi-O-Fe, or Bi-O-Bi and bond lengths as Bi-O, Bi-Fe, Bi-Fe that took place due to the internal stresses produced by Ba substitution [30].

**Figure 3:**
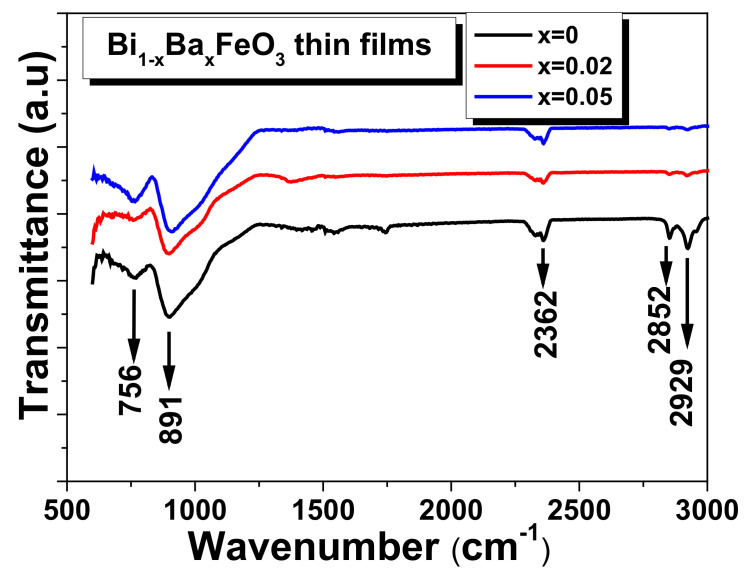
The room temperature FTIR spectra of Bi_1−x_Ba_x_FeO_3_ (0 ≤ x ≤ 0.05) thin films.

To complete the structural discussion, FTIR spectroscopy was employed to study the stretching and bending vibrations of the various bonds present in the studied films. The spectra of the BBFO films (with 0 ≤ x ≤ 0.05) in the IR (infrared) region were collected at room temperature in the range between 600 and 3000 cm^−1^ and are shown in the Figure 3. Since the measurements start from 600 cm^−1^, the FTIR spectra could not confirm exactly the Fe-O stretching or O-Fe-O bending vibration modes corresponding to the 547 cm^−1^ and 435 cm^−1^ respectively [35,36,37,38]. Anyway, the absorption band traces that can be seen around 600 cm^−1^ make us to assign it to the Fe-O stretching vibrations of FeO_6_ group in the perovskite compounds [35]. The obvious absorption bands near 756 cm^−1^ and ~891 cm^−1^ can be attributed to the Bi-O bending vibration for the pure BFO film [39] and also may be due to the metal-oxygen Ba-O stretching vibration [40] for BBFO films. The band located at ~2362 cm^−1^ most possibly indicates the presence of nitrate ions [41]. The peaks observed at ~2852 and 2900 cm^−1^ are assigned to C-H symmetric stretching vibrations [41]. The presence of metal-oxide bands in the measured spectrum indicates that the BBFO have crystallized in a perovskite structure for all the films. No significant peak shifting was observed due to the barium doping.

**Figure 4:**
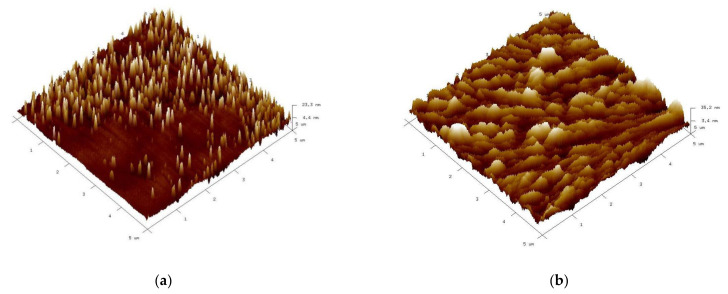

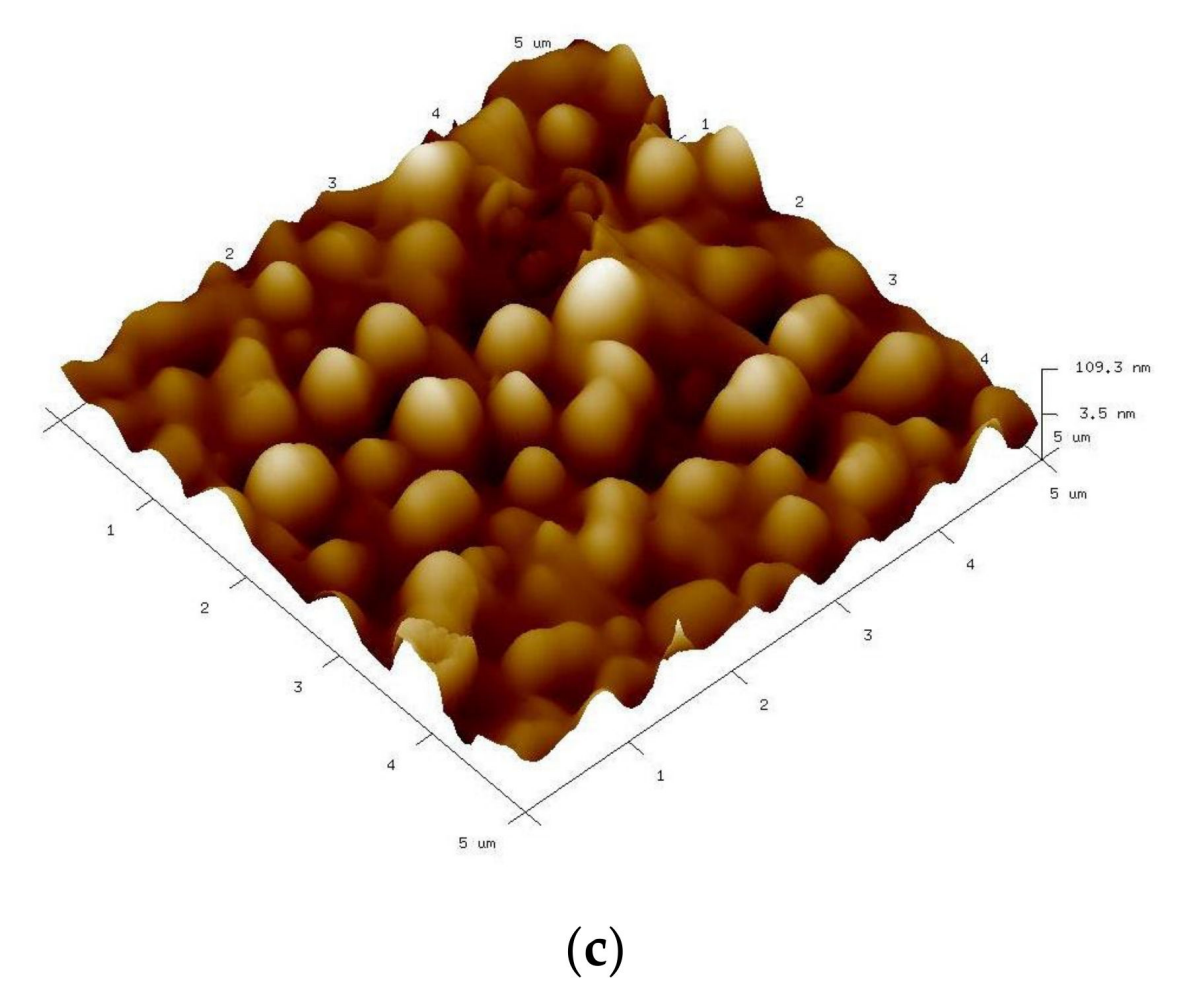
AFM images showing the surface topography 3D of the Bi_1−x_Ba_x_FeO_3_ thin films for: x = 0 (**a**), x = 0.02 (**b**), and x = 0.05 (**c**).

The surface morphology of the studied films was investigated by using atomic force microscopy (AFM) over 5 × 5 μm^2^ area in semi-contact mode. In the Figure 4 are presented the 3D topography AFM images for BBFO thin films with x = 0, 0.02, and 0.05 Ba^2+^ concentrations. It can be seen that all the films have grown without any cracks but in the same time the Ba addition affects the thin films microstructure and surface morphology. As Ba^2+^ concentration level increases, the grains tend to agglomerate and form larger particles that leads to surface inhomogeneity. From AFM morphology measurements it is evidenced an increase in the surface root mean square roughness (*R_rms_*) with increasing percentage of Ba^2+^ ions. The surface root mean square roughness varies from 4.9 nm to 8.9 nm and 31.8 nm, respectively for Ba^2+^ doping concentrations increasing from x = 0 to x = 0.05.

**Figure 5:**
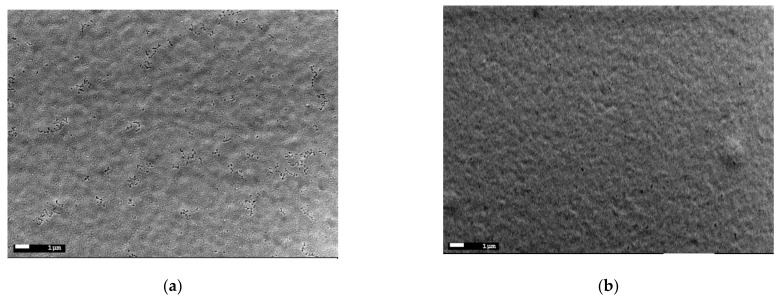

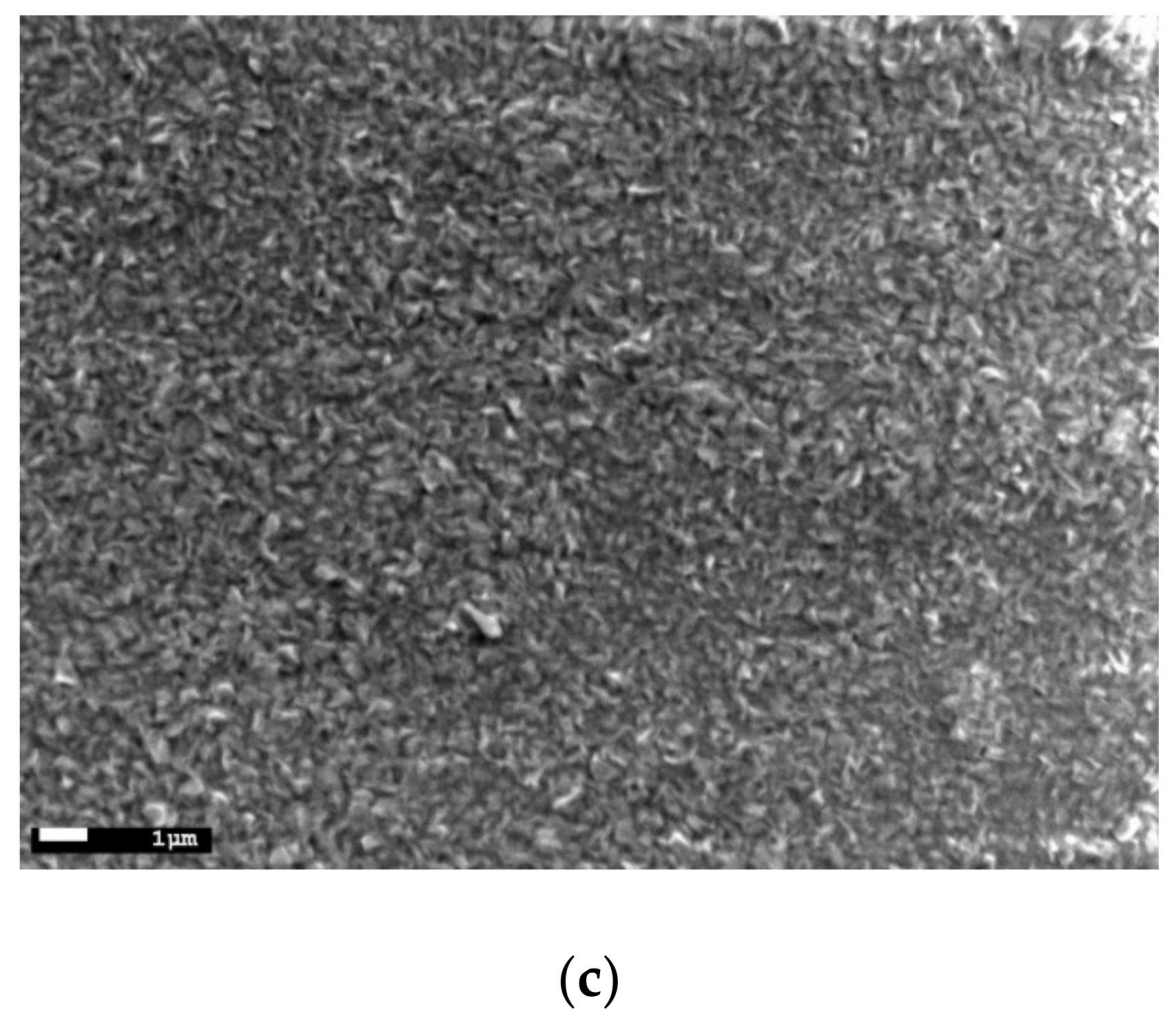
SEM images of the Bi_1−x_Ba_x_FeO_3_ thin films for: x = 0 (**a**), x = 0.02 (**b**), and x = 0.05 (**c**).

Figure 5 shows the surface SEM images of the BBFO thin films with x = 0, 0.02, and 0.05 concentrations. Since all other synthesis parameters are the same, it can be concluded that with increasing Ba^2+^ concentration, the morphology of the particle and grains changed. As shown in the Figure 5a, the pure BFO thin films present a smooth surface morphology with a small degree of porosity. With Ba doping increasing, the porosity decreases due to the grain size increasing that become irregular (as shape) for the x = 0.05 concentration, as it can be observed from Figure 5b,c. The average grain size of the films increases from ~100 nm for the pure BFO to ~500 nm for the Ba-doped BFO film with x = 0.05 concentration. Most probably, this is due to the fact that when Bi^3+^ is substituted by Ba^2+^ ions, it results in the formation of a higher number of oxygen vacancies for charge compensation and consequently in a change of nucleation rate and growth leading to particle coarsening. The present data are not in agreement with the reported data in Ref. [30] that found a decrease in the grain sizes with an increase in Ba due to the oxygen vacancies reduction that appears as an effect of charge compensation mechanism. An increase in grain size has been reported for similar BiFeO_3_ system doped with La^3+^ [41], NaNbO_3_ [42] or even with Ba^3+^ [43].

### 3.2. Optical Properties of BBFO Thin Films

**Figure 6:**
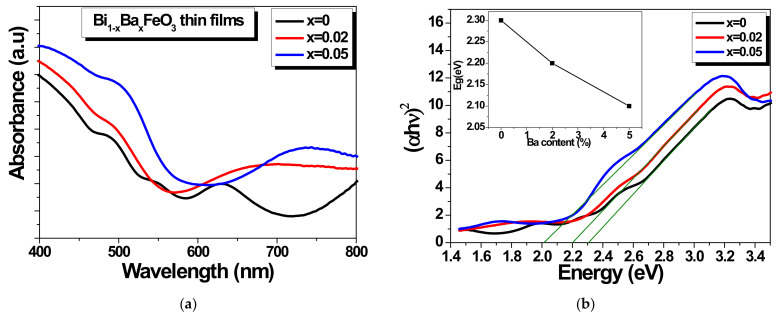
BBFO films optical properties: UV-vis absorption spectra (**a**). The calculation of the band gap energy of the BBFO films. Inset represents the band gap energy function of Ba concentration dependences (**b**).

The optical properties of the studied BBFO films were investigated by recording their UV-vis absorption spectra which are shown in the Figure 6. The optical absorption provides important information about the electronic states of the systems since the UV-vis absorption edge is in strong correlation with the band gap energy of the photocatalyst [44,45].

It can be observed from Figure 6a that there is absorption in the range of about 400–550 nm, which indicates that all BiFeO_3_-based films can absorb higher amounts of visible light. The optical measurements results suggest the potential applications of the Ba-doped BiFeO_3_ films as visible-light photocatalysts. To estimate the energy band gap of the films, the Tauc formula [44] was used, given by the Equation (3):

(3) $${\alpha h\nu =A(h\nu -E}_{g}{)}^{n/2}$$

where *α*, *h*, *ν*, *Eg*, *A,* and *n* represent the absorption coefficient, Planck constant, light frequency, band gap energy, proportional constant, and the power index that depend on the nature of transition. The value of *n* is 1 if considering that BiFeO_3_ is a direct band gap transition material [44]. The corresponding values of the *Eg* of BBFO films were estimated by extrapolating the linear part of the Tauc plots up to the (*αhν*)^2^ = 0 axis, as shown in the Figure 6b. According to Tauc relation, the estimated values for the energy band gap are 2.3 eV, 2.2 eV, and 2.1 eV for x = 0, 0.02, and 0.05 Ba doping concentrations. The bang gap estimated for BFO of ~2.3 eV is comparable with the values reported in the literature for BiFeO_3_-based systems [44,46,47]. Inset of the Figure 6b shows that the band gap is narrowed after doping with Ba ions and decreases gradually with the increase in the doping content, which is expected to result in an increase of photocatalytic response.

### 3.3. Photocatalytic Degradation of RhB

The photocatalytic degradation ability of the BBFO films was tested using RhB as an organic pollutant for degradation. The tests of BBFO films as photocatalysts were carried out by exposing the films to solar light for 6 h. The corresponding absorption spectra were recorded in the range of (350–600) nm. For comparison, a reference absorption test in the dark was registered before each experiment. The aqueous solution was chosen to have the pH value of ~7.

**Figure 7:**
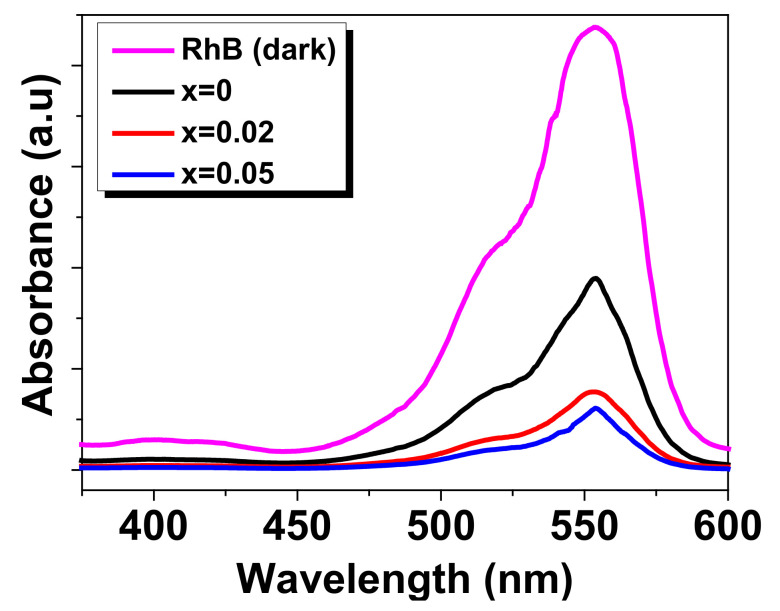
UV-vis absorbance spectra of photodegradation of the RhB solutions with BBFO thin films under solar light irradiation of 6 h.

Figure 7 shows the UV-vis absorbance curves of RhB during photocatalytic degradation.

It can be seen that Ba doping generally leads to a decrease of the absorbance of RhB in comparison to the non-irradiated reference demonstrating the photocatalytic activity of the studied films. As the concentration of Ba ions increases, the absorbance peak of the RhB degradation further decreases. The degradation efficiency (D) of RhB was determined using the following formula:

(4) $$D\left(\%\right)=\frac{A_{0}-A}{A_{0}}\times \;100$$

where *A*_0_ represents the initial absorbance of RhB solution measured at *λ_max_* = 553 nm and *A* is the absorbance after 6 h of solar light irradiation.

According to Figure 7, the calculated values for degradation efficiency for Ba-doped BFO thin films are: ~57% for x = 0, ~83% for x = 0.02, and ~86% for x = 0.05. It is obvious that the photocatalytic efficiency of BFO material was improved in the presence of Ba^2+^ ions for degradation of RhB. The higher degradation rate was obtained for the thin films with x = 0.05 Ba doping concentration since this film exhibits also the narrow band gap (2.1 eV) which results in the most efficient utilization of the sun light. It is known that the basic principle of semiconductor photocatalysis involves photogenerated electron-holes (e^−^-h^+^) pairs which react with the surface adsorbed oxygen or with water molecules to give reactive radicals leading to the decomposition of used pollutant [44,48]. The sunlight irradiation begins the photocatalytic reaction by absorbing the light (hν) energy which leads to the e^−^-h^+^ pairs generation that contributed to the formation of reactive radicals: (i) The e^−^ will react with the surface-adsorbed oxygen to generate superoxide radicals ($O_{2}^{.-}$); (ii) the h^+^ will react with water molecules to generate hydroxyl radicals (OH) [48]. Then, both hydroxyl radicals and superoxide play an important role in the degradation process of RhB. The present results can be explained by the fact that Ba doping leads to reducing of the recombination rate of photogenerated e^−^-h^+^ pairs (a better charge separation) which contribute to the formation of reactive radicals (their number increases with Ba addition increasing) and finally to the improving of photocatalytic response.

## 4. Conclusions

In the present work, it was investigated the influence of Ba doping on the structural, microstructural, optical properties, and photocatalytic activity of BFO thin films. Bi_1−x_Ba_x_FeO_3_ thin films were synthesized on glass substrates by using sol-gel-assisted spin-coating technique. The X-ray analysis revealed the formation of rhombohedral perovskite structure for all deposited films. FTIR results confirm that the BBFO have crystallized in a perovskite structure due to the presence of metal-oxide bands in the measured spectrum. The AFM morphology images evidenced an increase in the surface root mean square roughness with increasing percentage of Ba^2+^ ions from 4.9 nm for the pure BFO to 8.9 nm and 31.8 nm for x = 0.02 and x = 0.05 Ba doping concentrations, respectively. The optical measurements results suggest the potential applications of the Ba-doped BiFeO_3_ films as visible-light photocatalysts due to the narrowing of the band gap after the doping with Ba ions which decreases gradually with the increase in the doping content. The photocatalytic tests revealed that Ba doping of BFO material leads to the enhancement of photocatalytic response. Therefore, Ba-doped BiFeO_3_ thin films are good candidates for applications as visible-light photocatalysts.

## Figures and Tables

**Table 1:** Structural parameters calculated * for the Bi_1−x_Ba_x_FeO_3_ (0 ≤ x ≤ 0.05) thin films.

Samples	Crystal Symmetry	*a*(Å)	Cell Volume *V* (Å^3^)	Crystallite Size *D* (nm)	Tolerance Factor (*t*)
x = 0	Rhombohedral	3.9262	60.57	41.22	0.890
x = 0.02	Rhombohedral	3.9441	61.34	14.55	0.892
x = 0.05	Rhombohedral	3.9322	60.79	18.67	0.893

* by using the formula from Ref. [34].
